# Neuronal Deletion of *Tumor Susceptibility Gene 101* (*Tsg101*) Causes Rapid Apoptotic Loss of Hippocampal CA3 Neurons

**DOI:** 10.3390/biom15060786

**Published:** 2025-05-28

**Authors:** Will P. Walker, Megan Lea Ratz-Mitchem, Kay-Uwe Wagner, Teresa M. Gunn

**Affiliations:** 1 McLaughlin Research Institute, Great Falls, MT 59405, USA; meganratz46@gmail.com (M.L.R.-M.); 2 Department of Oncology, Center for Molecular Medicine and Genetics, Wayne State University, Detroit, MI 48202, USA; kuwagner@wayne.edu; 3 College of Osteopathic Medicine—Montana, Touro University, Great Falls, MT 59405, USA

**Keywords:** *Tumor Susceptibility Gene 101* (*Tsg101*/TSG101), endosomal trafficking, neurodegeneration, amyloid beta

## Abstract

Endosomal dysfunction is one of the earliest cellular signs in Alzheimer’s disease. Tumor susceptibility gene 101 protein (TSG101) is a component of the endosomal sorting complex required for transport (ESCRT)-I, which plays a key role in sorting ubiquitinated cell surface proteins and lipids onto intraluminal vesicles of multivesicular bodies for trafficking to lysosomes or autophagosomes for degradation, or to the plasma membrane for exosomal secretion. TSG101-dependent trafficking has been implicated in the propagation and spread of misfolded proteins associated with neurodegenerative diseases. We used transgenesis mice to study the in vivo consequences of disrupting TSG101-dependent trafficking in adult neurons. Mice lacking *Tsg101* in forebrain neurons (*Tsg101^ck2-null^*) showed rapid loss of hippocampal neurons and progressive forebrain atrophy. Astrogliosis was apparent in the dentate gyrus within 1 week of deleting *Tsg101*, followed by apoptosis of hippocampal CA3 neurons and accumulation of the autophagy adapter P62/SQSTM1 and ubiquitinated proteins. Failure to detect lipidated LC3 indicated autophagy was impaired rather than upregulated. Endosomal markers (RAB5 and RAB7) and amyloid protein also accumulated in hippocampal neurons of *Tsg101^ck2-null^* mice. Our data establish a critical role for TSG101 in neuronal survival and demonstrate the importance of the in vivo assessment of gene and protein functions.

## 1. Introduction

Alzheimer’s disease (AD) is the most common neurodegenerative disorder, with an estimated 55 million people worldwide currently living with AD. It is associated with the accumulation of extracellular amyloid β (Aβ) plaques and intracellular neurofibrillary tangles of hyperphosphorylated tau protein (p-tau), although one of the earliest cellular features is endosomal dysfunction in neurons [1,2,3,4]. Studies have detected abnormally enlarged early endosomes and elevated expression of the small guanosine triphosphatase (GTPase), Ras-related proteins RAB5 (early endosome marker), and RAB7 (late endosome marker) in hippocampal neurons in individuals with mild cognitive impairment or AD, as well as in post mortem brain samples from Downs syndrome with AD and the Dp16 mouse model of Downs syndrome (with 3 copies of the *amyloid precursor protein* gene (*App*)), and in human-induced pluripotent stem-cell-derived neurons engineered to carry familial AD-associated mutations [4,5,6]. Treating Dp16 mice with RAB5-specific antisense oligonucleotides normalized endosomal RAB activity and reversed AD-related phenotypes, including tau hyperphosphorylation and neurotrophin signaling deficits, supporting a direct role for RAB5 dysfunction in AD pathogenesis [7]. In multiple cell types from AD patients or mouse AD models, endocytic activation associated with increased RAB5 activity led to late endosomal dysfunction [8,9], which suggests that increased RAB5 activity could in turn lead to elevated RAB7 expression. RAB5 is aberrantly activated by APP-βCTF (secretase-cleaved C-terminal fragment (CTF) of amyloid precursor protein (APP)), and the transgenic expression of activated RAB5 in mouse neurons led to the enlargement and mistrafficking of early endosomes, as well as synaptic plasticity deficits, tau hyperphosphorylation, and cognitive deficits [8,10,11]. Thus, endosomal defects may play a direct role in AD pathogenesis.

Tumor susceptibility gene 101 (TSG101) is a housekeeping gene that encodes a multidomain protein with many functions in different cellular compartments. TSG101 has been proposed to play roles in transcriptional regulation, cell proliferation, cell division, retroviral particle assembly, and viral budding, although it is best characterized as a component of the endosomal sorting complex required for transport (ESCRT)-I [12,13,14,15,16,17,18,19,20,21,22]. The ESCRT machinery sorts ubiquitinated proteins onto intraluminal vesicles (ILVs) of multivesicular bodies (MVBs) for trafficking either to the lysosome, for degradation, or to the plasma membrane, whereupon ILVs are secreted as a subclass of nanosized extracellular vesicles (EVs) called exosomes [23,24,25]. The cellular mechanisms that determine whether MVBs traffic their ubiquitinated cargoes to the lysosome or the plasma membrane remain poorly understood. APP is ubiquitinated and sorted through the endosomal system in an ESCRT-dependent manner [26,27,28,29], and *Tsg101* depletion in cultured cells inhibited the sorting of APP onto ILVs and to the lysosome, resulting in the intracellular accumulation of Aβ and a concomitant decrease in Aβ secretion [27].

There are many additional links between the endo-lysosomal pathway and AD. The AD risk genes identified in genome-wide association studies include loci that encode components of the endocytic trafficking pathway [30,31,32,33,34]. APP cleavage to form amyloid-β (Aβ) and CTFs is believed to occur, at least in part, in early endosomes [35,36,37,38], and the acidic environment of late endosomes or lysosomes would promote the self-assembly of Aβ peptides into fibrils that seed plaque formation when released from the cell, perhaps via exosomes or other EVs [39,40,41]. Neuronal cells in the brains of human AD patients and mouse and rat AD models, as well as in cell culture, have been shown to accumulate aggregation-prone and oligomerized Aβ peptides in MVBs and to secrete exosomes containing full-length APP, as well as CTFs and Aβ [42,43,44,45,46,47]. Neurally derived exosomes isolated from the blood of AD patients were reported to contain significantly higher levels of lysosomal proteins (cathepsin D and LAMP-1) and ubiquitinated proteins, indicative of endo-lysosomal dysfunction [48]. ESCRT components were associated with amyloid plaques in AD transgenic mice [41]. The knockdown of ESCRT proteins, including TSG101, promoted tau aggregation [49], and endosomally derived exosomes contain and may propagate the spread of p-tau [45,50,51,52,53].

Most studies on the molecular, biochemical, and intracellular functions of TSG101 have emphasized the relevance of their specific findings to diseases such as cancer and neurodegeneration, although the proposed mechanisms are rarely validated in genetically defined, in vivo disease models or primary human tissue samples. Consequently, the biological significance of the multifaceted functions of TSG101 in normal development, differentiation, and tissue homeostasis, as well as the potential roles of TSG101-mediated pathways in disease pathogenesis, are poorly defined [23]. In the present study, we used tamoxifen-induced conditional mutagenesis to delete *Tsg101* from forebrain neurons in adult mice. The neuronal deletion of *Tsg101* resulted in reactive astrogliosis in the dentate gyrus starting within 1 week of *Tsg101* deletion, followed by apoptosis of hippocampal CA3 neurons and forebrain atrophy. No upregulation of autophagy was detected. Impaired endosomal trafficking was indicated by the accumulation of ubiquitinated proteins, RAB5, and RAB7, and there was an increase in intracellular APP or its proteolytic derivatives in hippocampal neurons. This study supports the hypothesis that disrupted ESCRT function affects the trafficking of APP and its proteolytic derivatives, such as Aβ, and demonstrates that impairing TSG101-dependent processes leads to rapid-onset neurodegeneration in brain areas impacted in AD.

## 2. Materials and Methods

### 2.1. Mice

The animals were housed under standard conditions in the Animal Resource Center at the McLaughlin Research Institute. *Tsg101^tm1-Kuw^* (*Tsg101^fl^*) conditional knockout mice, which were created and maintained on a 129/SvJ genetic background, have been described previously [20]. As this allele has loxP sites 3kb upstream and 230 bp downstream of the first coding exon of *Tsg101*, Cre recombinase excises the proximal promoter region and first exon of Tsg101, resulting in a null allele. The *Tsg101^fl/fl^* mice were crossed to the tamoxifen-inducible Cre transgenic line, B6.FVB-Tg(Camk2a-cre/ERT2)2Gsc/Ieg (referred to as Camk2a-cre/ERT2), which expresses CreERT2 in forebrain neurons [54]. The Camk2a-cre/ERT2 mice were obtained as frozen embryos from the European Mutant Mouse Archive, Munich, Germany (EMMA ID EM:02125), and rederived by the McLaughlin Research Institute Transgenic Facility (RRID:SCR_023108). Cre-positive F1’s were backcrossed to *Tsg101^fl/fl^* animals to produce *Tsg101^fl/fl^* and *Tsg101^fl/+^* mice segregating for the Cre transgene. Tamoxifen-treated *Tsg101^fl/+^*; *Camk2a-cre/ERT2(+)* or *Tsg101^fl/fl^* without the Camk2a-cre/ERT2 transgene, or vehicle-treated *Tsg101^fl/fl^–Camk2a-cre/ERT2(+)* were used as controls, and no significant differences in brain pathology were observed between these groups. The brains of male and female mice were examined and no differences were detected between them. Despite the mixed genetic background, we also detected no gross differences in brain pathology between animals within the same genotype or treatment group. All animal procedures adhered to the Association for Assessment and Accreditation of Laboratory Animal Care guidelines and were approved by the Institutional Animal Care and Use Committee of the McLaughlin Research Institute (protocol TMG-80, approved 21 July 2009).

### 2.2. Genotyping

The animals were genotyped by PCR using GoTaq Green 2X Master Mix (Promega Corporation, Madison, WI, USA). The *Tsg101* genotyping primers were: Tsg101^wt^ GTTCGCTGAAGTAGAGCAGCCAG and CATTTCTGGAGTCCGATGCGCAG; Tsg101^fl^ AGAGGCTATTCGGCTATGACTG and TTCGTCCAGATCATCCTGATC; Tsg101^null^ GATGGTCATACCTGGTTAGAAAGC and CATTTCTGGAGTCCGATGCGCAG. The Camk2a-cre/ERT2 transgene was detected using the forward primer GGTTCTCCGTTTGCACTCAGGA and reverse primer GCTTGCAGGTACAGGAGGTAGT.

### 2.3. Activation and Verification of Cre Recombinase Activity

*Tsg101* deletion was induced at 12 weeks of age via the twice-daily intraperitoneal injection of 0.025 mg of tamoxifen (Sigma-Aldrich, St. Louis, MO, USA) per kg of body weight for three consecutive days, except for the oldest cohort (brains collected 140 days post-injection), which received twice-daily injections of 0.025 mg/kg for five consecutive days. The tamoxifen-treated *Tsg101^fl/fl^*–Camk2a-cre/ERT2(+) mice are referred to hereafter as *Tsg101^ck2-null^*. The tamoxifen was dissolved to 5 μg/mL in a 1:9 mixture of absolute ethanol and pharmacological-grade sunflower oil (Sigma-Aldrich). The control animals received injections of the 1:9 ethanol/oil vehicle mixture without tamoxifen. Every cohort collected contained tamoxifen-injected experimental and control animals as well as non-injected controls. As *Tsg101* deletion caused weight loss in *Tsg101^fl/fl^–Camk2a-cre/ERT2(+)* mice, the mice were monitored daily following tamoxifen treatment, and excessive weight loss (≥20% of initial body weight) was used as one of the humane end-point criteria. A liquid dietary supplement (Ensure Plus or Pet-Ag Esbilac) was provided to the treated animals as needed to support the maintenance of a healthy body weight.

The Cre recombinase-mediated deletion of the floxed *Tsg101* sequence was verified via PCR using the genotyping primers described above and a template of 10 ng genomic DNA isolated from dissected brain regions of 3 vehicle-injected and 3 tamoxifen-injected animals (2 weeks post-treatment), as well as from brains of 3 Camk2a-cre/ERT2-negative animals as a further negative control. Reductions in TSG101 protein levels were confirmed via the immunoblotting of brain lysates isolated two weeks after the tamoxifen treatment (*n* = 3 per genotype/treatment), using rabbit anti-TSG101 (Cat# 14497-1-AP, RRID:AB_2208090, Proteintech Group, Inc., Rosemont, IL, USA). The brain lysates were prepared by homogenizing brain tissue in solubilization buffer (50 mM Tris-HCl pH 8.0, 1 mM EDTA, 1% Igepal CA-630) containing cOmplete Protease Inhibitor Cocktail (Roche, Indianapolis, IN, USA). The blots were imaged using ECL Plus chemiluminescent substrate (Thermo Scientific Pierce, Rockford, IL, USA).

### 2.4. Histology

The brains were fixed in formalin, processed for histology via standard methods, and embedded in paraffin. The experimental and control brains were processed together in each batch, with care taken to avoid long exposure to 70% ethanol, as this can cause artifactual vacuolation in rodent nervous tissue [55]. The paraffin-embedded brains were sectioned at 3 or 5 mm and stained with hematoxylin and eosin (H&E). The following numbers of *Tsg101^ck2-null^* brains were examined using H&E for each post-injection time-point: 1 week = 4; 2 weeks = 15; 4 weeks = 3; 6 weeks = 4; 8 weeks = 3; 20 weeks = 2 (only two *Tsg101^ck2-null^* mice survived this long). Equal or greater numbers of controls were also examined at each time-point. A sample size of 3 is expected to identify a statistically significant different (*p* < 0.05) as long as at least 90% of animals show a consistent phenotype. The individuals performing and analyzing the experiments were not blinded to genotype. Immunohistochemistry (IHC) was performed on paraffin sections (*n* = 3 per genotype/antibody/time-point examined), which were pre-treated for antigen retrieval using 10 mM of sodium citrate (pH 6.0, 100 C, 10 min). The antibody information is provided in Section 2.6. Immunolabelling was detected using fluorescent secondary antibodies (Vector Labs, Newark, CA, USA), horseradish peroxidase (HRP)-conjugated secondary antibodies (anti-rabbit IgG-peroxidase, Sigma-Aldrich Cat# A0545, RRID:AB_257896), or HRP-conjugated avidin and biotinylated secondary antibodies of the appropriate isotype (Vector Labs, Newark, CA, USA) and the peroxidase substrate diaminobenzidine (DAB) (Trevigen Inc., Gaithersburg, MD, USA), NovaRed (Vector Labs), or both. The sections were counterstained with hematoxylin. For all studies, the experimental and control samples were processed together to eliminate inter-experimental variability, and at least 3 independent samples were examined for each genotype. The stained sections were imaged on a Zeiss AxioImagerM1 microscope using a A623C color camera (PixeLink, Ottawa, ON, Canada) or on a Fluoview 1000 confocal microscope (Olympus Corporation, Center Valley, PA, USA).

### 2.5. Immunoblotting

For Western blotting, the brain tissue was homogenized in RIPA lysis buffer (50 mM of Tris, 150 mM of NaCl, 1% NP40, 0.1% sodium deoxycholate) supplemented with cOmplete Protease Inhibitor Cocktail (Roche). The cellular debris was pelleted via centrifugation and the supernatant was diluted in 2X SDS loading buffer (0.125 M Tris-HCl, 4% SDS, 20% glycerol, and 0.01% bromophenol blue, 10% 2-mercaptoethanol). The proteins were electrophoresed through 8% or 12% SDS–polyacrylamide gels and transferred to an Immobilon P membrane (Millipore Corporation, Burlington, MA, USA). The immunoblotting was performed following standard protocols. Briefly, the membranes were blocked with 5% non-fat dry milk in Tris-buffered saline with Tween 20 (TTBS). The antibodies were diluted in TTBS and HRP-conjugated secondary antibodies (goat anti-rabbit IgG-peroxidase, Cat# A0545, RRID:AB_257896, Sigma-Aldrich; or goat anti-mouse IgG, Cat# 554002, RRID:AB_395198, BD Biosciences, Franklin Lakes, NJ, USA) and imaged using Clarity ECL Western Blotting Substrate (BioRad Laboratories, Hercules, CA, USA) and either a Bio-Rad ChemiDoc imaging system or the rapid capture setting on an Azure 300 Imager (Azure Biosystems Inc., Dublin, CA, USA). For LC3 western, the Azure software used to verify the signal was not saturated, then bands were quantified using AzureSpot Pro v.1.0 (Azure Biosystems). The antibody information is provided in Section 2.6.

### 2.6. Antibodies

The primary antibodies used were: rabbit anti-cleaved caspase-3 (Cat# 2305-PC-100, RRID:AB_2665453, Trevigen), mouse anti-NeuN (Cat# MAB377, RRID:AB_2298772, Millipore), rabbit anti-GFAP (Cat# 16825-1-AP, RRID:AB_2109646, Proteintech Group), mouse anti-mono and polyubiuquitinated conjugates (Cat# BML-PW8810, RRID:AB_10541840, Enzo Life Sciences, Farmingdale, NY, USA), rabbit anti-RAB5 (LS-C138527, now sold by Abcam, Cat# ab109534, RRID:AB_10865740, LifeSpan Biosciences, Seattle, WA, USA), guinea pig anti-p62 (Cat# 03-GP62-C, RRID:AB_1542690, ARP American Research Products, Waltham, MA, USA), rabbit anti-LC3A (Cat# NB100-2331, RRID:AB_10001955, Novus International, Chesterfield, MO, USA), rabbit anti-LC3 (RRID:AB_2716621, a gift from Dr. Masahiro Shibata), mouse anti-RAB7 (Cat# ab58029, RRID:AB_945127, Abcam Limited, Waltham, MA, USA), and mouse monoclonal 4G8 (Cat#800701, RRID:AB_2564633, BioLegend, San Diego, CA, USA), which is reactive to amino acids 17-24 of Aβ and to APP. For the immunoblotting, mouse anti-beta-tubulin-III (3F3-G2) (Cat# sc-53140, RRID:AB_793543, Santa Cruz Biotechnology, Dallas, TX, USA) was used as a loading control.

### 2.7. Statistics

Brain weight, body weight, food consumption, and protein levels were assessed for statistically significant differences using Student’s *t*-test, and F tests were used to compare variances, using Prism 10 analysis software (GraphPad Software, San Diego, CA, USA). The minimum sample sizes were selected to provide at least 90% power of detecting a significant difference (*p* = 0.05) if at least 90% of the animals within a genotype or treatment group gave a consistent phenotype or result. As some of the *Tsg101^ck2-null^* mice were lost within 14 days of *Tsg101* deletion, an excess number of *Tsg101^fl/fl^–Camk2a-cre/ERT2(+)* mice were treated with tamoxifen to collect the desired number of mice at later time-points.

## 3. Results

**Figure 1:**
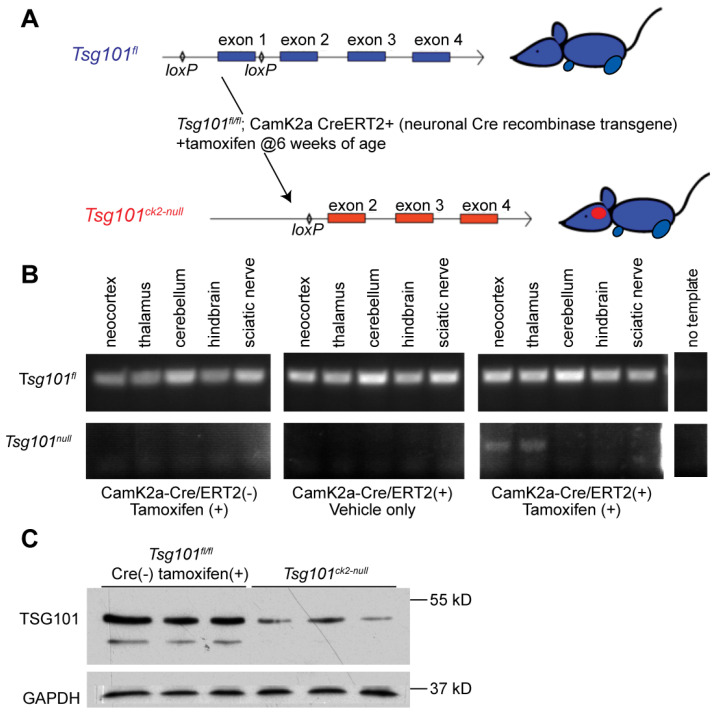
Generation and validation of the *Tsg10^ck2-null^* allele. (**A**) Schematic representation of the 5′ region of the *Tsg101^fl^* conditional allele, showing loxP sites flanking exon 1 (top, in blue) and deletion of exon 1 in mice expressing the Camk2a-cre/ERT2 transgene following tamoxifen treatment (bottom, in red). (**B**) Allele-specific PCR results from amplification of the *Tsg101^fl^* (top) or *Tsg101^ck2-null^* allele (bottom) from DNA isolated from indicated tissues dissected from Camk2a-cre/ERT2 transgenic and non-transgenic *Tsg101^fl/+^* animals two weeks after administration of tamoxifen or oil. Results shown are representative of 4 replicates. (**C**) Western blots of forebrain lysates from 3 *Tsg101^ck-null^* mice and 3 controls (*Tsg101^fl/fl^* without the Camk2a-cre/ERT2 transgene), showing a reduction in TSG101 protein levels in Cre(+) brain tissues 2 weeks after the initiation of tamoxifen treatment.

**Figure 2:**
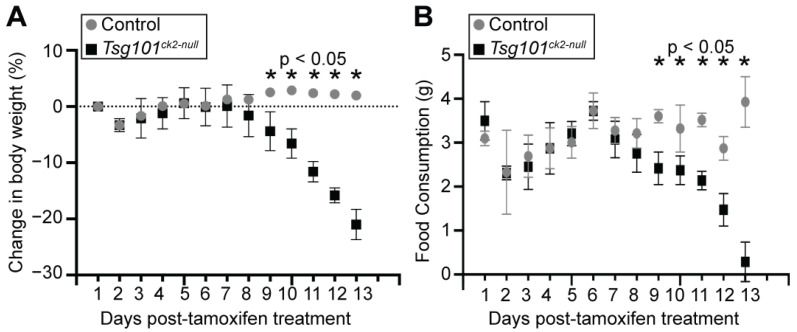
Effects of neuronal ablation of *Tsg101* on body weight and food intake: (**A**) *Tsg101^ck2-null^* mice showed a significant decline in body weight starting 9 days after tamoxifen treatment; (**B**) *Tsg101^ck2-null^* mice also showed a significant decline in food consumption starting 9 days after tamoxifen treatment. Statistically significant timepoints are indicated by asterisks.

*Tsg101* was ablated in forebrain neurons of adult *Tsg101* conditional knockout mice carrying the Camk2a-cre/ERT2 transgene by treating them with tamoxifen at 12 weeks of age (Figure 1A). This Cre transgene has previously been shown to have low activity in the hippocampus in the absence of tamoxifen and robust activity, especially in neurons of the cortex, dentate gyrus, and hippocampus upon tamoxifen treatment [54]. Initially, *Tsg101^fl/fl^–Camk2a-cre/ERT2+* and control (*Tsg101^fl/fl^*, *Camk2a-cre/ERT2-,* and *Tsg101^fl/-^*; *Camk2a-cre/ERT2+*) mice were given twice-daily IP injections of tamoxifen or vehicle control (1:9 ethanol/sunflower oil) for 5 days. The deletion of *Tsg101* in the tamoxifen-treated mice was verified via PCR on DNA isolated from dissected regions of the CNS and the sciatic nerve using primers that distinguish the endogenous wild-type allele, the floxed allele, and the recombined (deleted) allele (Figure 1B). Immunoblotting confirmed a reduction in TSG101 protein levels in the brains of tamoxifen-treated *Tsg101^fl/fl^*; *Camk2a-cre/ERT2+* mice, referred to hereafter as *Tsg101^ck2-null^* (Figure 1C). In initial studies, these mice showed significant, progressive weight loss and reduced food intake relative to controls, starting 9 days after the initiation of tamoxifen treatment (Figure 2A,B). By 13–14 days post-tamoxifen, the Tsg101^ck2-null^ mice lost ≥ 20% of their body weight and were humanely euthanized. Based on home cage observation, the mice would stare blankly into space for periods of time, and they appeared to generally lose interest in even trying to eat. The tamoxifen treatment regime was reduced to 2 injections per day for 3 consecutive days, and the *Tsg101^ck2-null^* mice were offered a pipette of liquid Ensure meal replacement or Esbilac supplement every 3–4 h for ~2 weeks, starting 8 days after the initiation of tamoxifen injections. Under these conditions, the anorexia and weight loss experienced by the *Tsg101^ck2-null^* mice appeared to be transient, as after liquid oral supplementation, most of the mice resumed appropriate feeding behavior and weight gain, although some still appeared frail and had a reduced lifespan. As the brain histology did not discern any differences between the *Tsg101^ck2-null^* mice generated using either tamoxifen treatment regime, the samples were used interchangeably.

**Figure 3:**
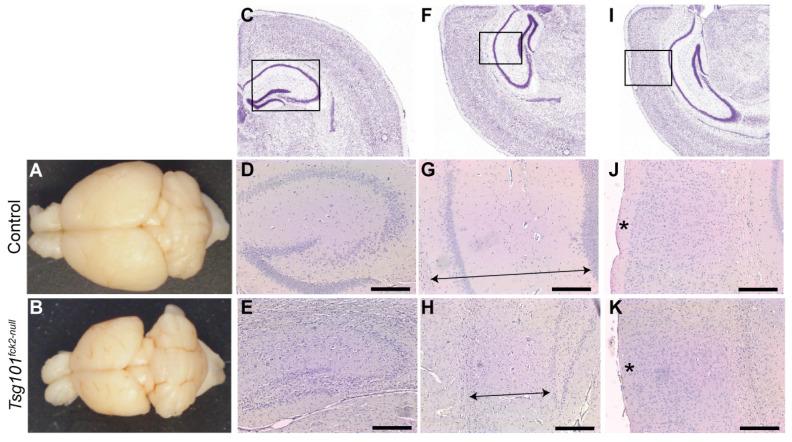
Significant neurodegeneration occurs within 20 weeks of deleting *Tsg101* from forebrain neurons. (**A**,**B**) A gross analysis of brains from tamoxifen-treated control (**A**) and *Tsg101^ck2-null^* (**B**) mice demonstrates severe neocortical atrophy. (**C**–**H**) Histological assessment of the hippocampus shows significant loss of pyramidal neurons in the hippocampus and dentate gyrus (**E**), shrinkage of the hippocampus (distance indicated by arrows in (**G**,**H**)), and reduction in the thickness of cortical layer 1 (indicated by * in (**J**,**K**)) in *Tsg101^ck2-null^* brains relative to controls ((**D**,**G**,**J**), respectively). Panels (**C**,**F**,I) are from the Allen Mouse Brain Atlas [56] and the boxed areas indicate the regions shown at higher magnification in (**D**,**E**), (**G**,**H**), and (**J**,**K**), respectively. Scale bars: 500 µm.

**Figure 4:**
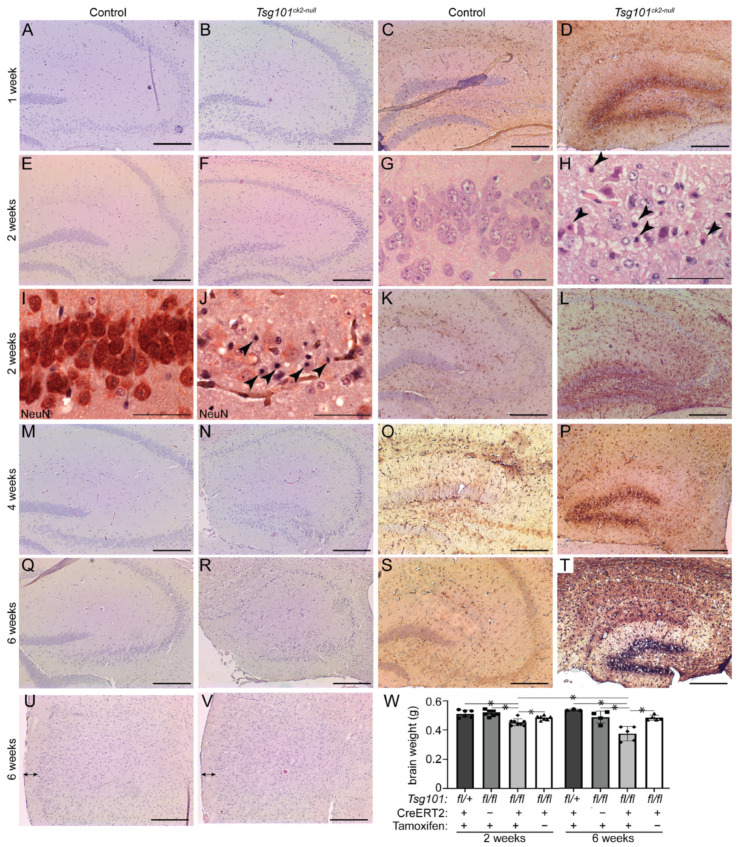
*Tsg101^ck2-null^* brains show progressive loss of hippocampal neurons associated with reactive astrogliosis in the dentate gyrus. (**A**–**T**) Hippocampal sections from control and *Tsg101^ck2-null^* brains (as specified above each column) were H&E-stained (**A**,**B**,**E**–**H**,**M**,**N**,**Q**,**R**) or subjected to IHC staining for GFAP (**C**,**D**,**K**,**L**,**O**,**P**,**S**,**T**) or NeuN (**I**,**J**) 1, 2, 4, and 6 weeks after mice were given tamoxifen (as indicated to the left of each row). (**G**,**H**) Pyknotic nuclei (arrowheads in **H**) were present in the CA3 region 2 weeks after *Tsg101* deletion was induced by tamoxifen. (**I**,**J**) Loss of pyramidal neurons and the presence of pyknotic nuclei (arrowheads in **J**) were apparent in the CA3 region of *Tsg101^ck2-null^* mice at the 2-week time-point. (**U**,**V**) H&E staining of the cortex indicated no difference in cortical layer 1 thickness by the 6-week time-point. (**W**) Whole-brain weight of *Tsg101^ck2-null^* and control mice 2 and 6 weeks after tamoxifen (+) or vehicle (−) injection. Statistically significant (*p* < 0.05) comparisons are indicated by asterisks. Scale bars: 500 µm (**A**–**F**,**K**–**V**); 50 µm (**G**–**J**).

Twenty weeks after the tamoxifen treatment was initiated, the *Tsg101^ck2-null^* mice showed a substantial reduction in forebrain size, apparent even upon gross inspection (Figure 3A,B). Hematoxylin and eosin (H&E)-stained coronal sections revealed marked reductions in the volume of the hippocampal formation (Figure 3C–H) and cortex layer 1 (Figure 3I–K). The granule cell layers of the dentate gyrus and hippocampus were much thinner in the *Tsg101^ck2-null^* brains (Figure 3C–H), consistent with loss of neurons. Since no other gross differences were observed in the cortex, nor in the thalamus or hypothalamus, our subsequent analyses focused predominantly on the hippocampal formation. To investigate the time course of neurodegeneration, *Tsg101^ck2-null^* and control brain sections were examined 1, 2, 4, and 6 weeks following tamoxifen treatment, using H&E staining to assess the overall morphology and IHC for glial fibrillary acidic protein (GFAP) to detect reactive astrogliosis. One week after tamoxifen treatment, the *Tsg101^ck2-null^* brains showed a small number of neurons with pyknotic nuclei in the CA3 subfield of the hippocampus and elevated GFAP immunoreactivity surrounding the granule cell layer of the dentate gyrus, indicative of reactive astrogliosis. The latter suggests that neuronal homeostasis was disrupted in the dentate gyrus, despite no overt neuron loss (Figure 4A–D). Within 2 weeks of tamoxifen treatment, the *Tsg101^ck2-null^* brains showed extensive loss of NeuN-positive neurons in the CA3 subfield of the hippocampus, associated with the presence of pyknotic nuclei (Figure 4E–J). Astrogliosis was still concentrated in the dentate gyrus, with weaker GFAP staining present more broadly throughout the hippocampal formation (Figure 4K,L). By 4 weeks after tamoxifen treatment, the pyramidal layers of all regions of the hippocampus, as well as the dentate gyrus, were thin and showed the presence of pyknotic nuclei (Figure 4M,N), and GFAP staining remained strongest in the dentate gyrus (Figure 4O,P). By the 6-week time-point, only a thin layer of neurons remained in the granule cell layers of the hippocampus and dentate gyrus (Figure 4Q,R). At this time-point, astrogliosis was observed throughout the hippocampal formation and the dentate gyrus continued to show stronger GFAP staining than in other regions (Figure 4S,T). Although cortex layer 1 was significantly thinner in the brains of the *Tsg101^ck2-null^* mice 20 weeks after tamoxifen treatment, no difference was observed up to the 6-week time-point (Figure 4U,V). Consistent with a significant loss of neurons, the brains of the *Tsg101^ck2-null^* mice weighed significantly less than those of the control mice by the 2-week time-point (Figure 4W) and atrophy was progressive, as indicated by the significant reduction in brain weight of the *Tsg101^ck2-null^* mice between the 2- and 6-week time-points (Figure 4W). By 6 weeks post-tamoxifen, there was a ~20% reduction in total brain mass in *Tsg101^ck2-null^* mice relative to controls.

**Figure 5:**
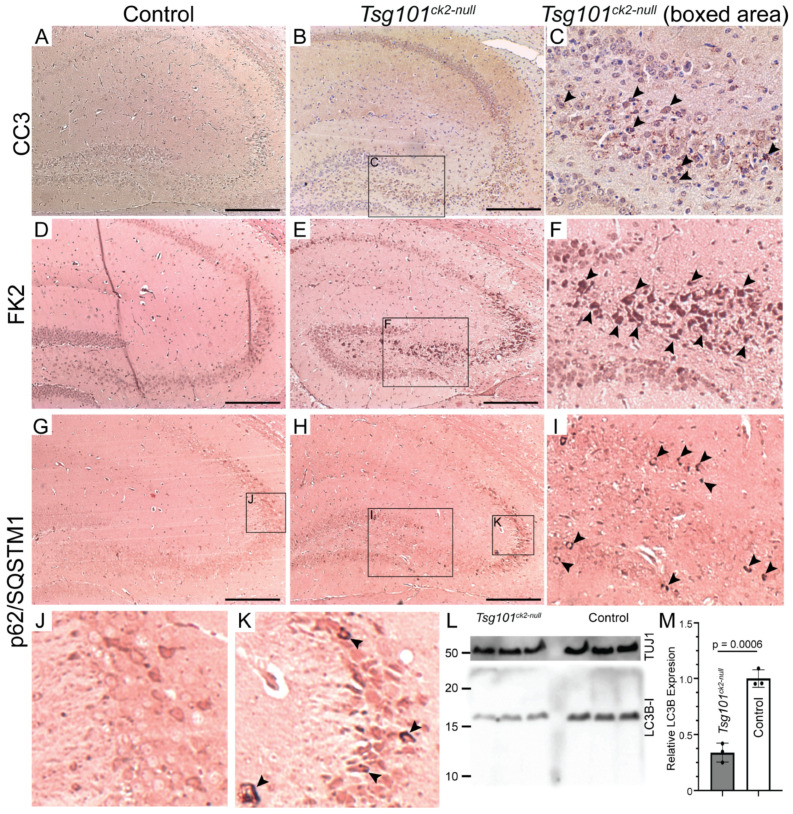
Characterization of ubiquitination and cell death pathways in *Tsg101^ck2-null^* hippocampi 2 weeks after tamoxifen treatment. (**A**–**C**) IHC for activated cleaved caspase 3 (CC3) identified accumulation CC3-positive punctae in nuclei (arrowheads) of *Tsg101^ck2-null^* hippocampal neurons. Boxed area in (**B**) is shown at higher magnification in (**C**). (**D**–**F**) Ubiquitinated proteins were detected via IHC using the FK2 antibody. *Tsg101^ck2-null^* hippocampal neurons displayed elevated staining relative to controls, consistent with the accumulation of ubiquitinated inclusions. Area boxed in (**E**) is shown at higher magnification in (**F**), with arrowheads indicating some of the cells in CA3 that demonstrate strong FK2 staining. (**G**–**K**) IHC for p62/SQSTM1 revealed accumulation of p62 (arrowheads in (**I**,**K**)) in CA3 neurons of *Tsg101^ck2-null^* mice. Boxed area in G is shown at higher magnification in (**J**). Boxed areas in (**H**) are shown at higher magnification in (**I**,**K**). (**L**,**M**) Hippocampi dissected from *Tsg101^ck2-null^* and control mice 1 week after tamoxifen treatment was initiated were subjected to Western blotting for LC3-II (LC3B) and TUJ1. A band consistent in size with LC3B-I but not LC3B-II was detected, and when normalized against TUJ1, was significantly reduced in *Tsg101^ck2-null^* hippocampi, *p* = 0.0002. Scale bars: 500 µm.

Degenerating pyramidal cells in the CA3 region of the hippocampus of *Tsg101^ck2-null^* mice showed morphological and immunohistochemical evidence of apoptosis, such as an abundance of pyknotic nuclei (Figure 4H,J) and expression of the cleaved (active) form of the apoptosis effector caspase-3 (CC3) (Figure 5A–C). CA3 pyramidal cells also showed increased staining for mono- and poly-ubiquitinated proteins (Figure 5D–F), consistent with impaired sorting of ubiquitinated proteins into MVBs, a process that includes substrate deubiquitination. As loss of TSG101 function has been reported to upregulate autophagy and dysregulated autophagy contributes to caspase-dependent neuronal apoptosis in cultured cells [57,58], we examined the expression of the autophagy adapter protein, p62/SQSTM1 (Sequestosome-1), as well as LC3 (microtubule-associated protein 1A/1B-light chain 3), which undergoes conversion from LC3-I to LC3-II via the addition of a phosphatidylethanolamine group to the C terminus prior to being recruited to autophagosomes. In *Tsg101^ck2-null^* brains collected 1 week following the initiation of tamoxifen treatment, SQSTM1-positive punctae were observed in some pyramidal cells in the dentate gyrus, and more frequently in the hippocampal CA3 region (Figure 5G–K). As SQSTM1 is degraded through autophagy, elevated levels could indicate impairment of this pathway. IHC for LC3B did not reveal punctae of staining in *Tsg101^ck2-null^* brains (data were negative, not shown here). Immunoblotting indicated reduced expression of LC3-I in *Tsg101^ck2-null^* hippocampi but a band consistent in size with LC3-II was not detected, even on long exposures or on blots of subcellular fractions expected to contain autophagosomes, prepared as described by Lai et al. [59] (Figure 5L,M and data were negative, not shown here). This result was replicated using two different antibodies against LC3. The reduced expression of LC3-I was unlikely to be due to a loss of neurons, as the expression was normalized against a neuron-specific marker, TUJ1. Taken together, these data suggest that either autophagy was not upregulated in *Tsg101^ck2-null^* neurons, or that it was a very early and transient effect, since no LC3-II could be detected within one week of the induction of *Tsg101* deletion.

**Figure 6:**
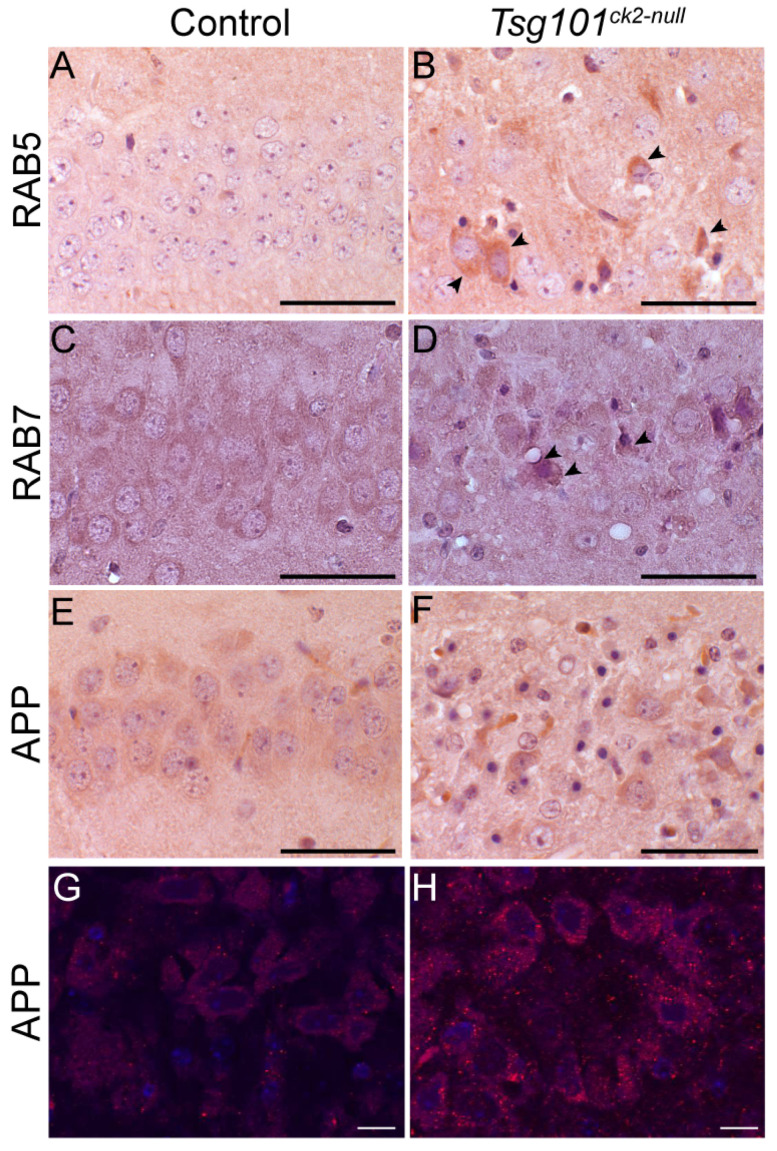
Endosomal markers and intracellular APP accumulated in hippocampal neurons within 2 weeks of deleting *Tsg101* from forebrain neurons. (**A**–**D**) *Tsg101*^ck2-null^ hippocampal CA3 neurons accumulated early (RAB5, **A**,**B**) and late (RAB7, **C**,**D**) endosomal markers. Arrowheads in (**B**,**D**) indicate representative cells with elevated RAB5 or RAB7 expression, respectively. (**E**–**H**) IHC with the 4G8 antibody against APP (which also detects Aβ) showed more intense staining in *Tsg101*^ck2-null^ hippocampal CA3 neurons relative to controls (**E**,**F**). Immunofluorescence staining revealed more 4G8-positive punctae in *Tsg101*^ck2-null^ CA3 neurons relative to controls (**G**,**H**). Images shown in (**G**,**H**) were captured from slides stained at the same time, using the 40× objective and identical PMT voltage settings on an Olympus Fluoview 1000 confocal microscope, and are representative of 4 independent samples for each genotype. Scale bars (**A**–**F**): 50 µm; scale bars (**G**,**H**): 10 µm.

Our mouse model provided the opportunity to examine, in vivo, whether the neuronal depletion of *Tsg101* leads to endosomal defects similar to those observed in AD brains. IHC on coronal sections from *Tsg101^ck2-null^* and control brains collected 2 weeks post-tamoxifen revealed the accumulation of RAB5 and RAB7 in hippocampal CA3 neurons (Figure 6A–D). Staining using an antibody reactive to APP amino acids 17–24 (clone 4G8), thereby recognizing APP and Aβ, revealed stronger staining in *Tsg101^ck2-null^* hippocampal CA3 neurons relative to the controls (Figure 6E,F). Immunofluorescence staining with clone 4G8 followed by confocal imaging revealed more abundant intraneuronal puncatae, consistent with impaired trafficking of APP/Aβ.

## 4. Discussion

The results of this study demonstrate that deleting *Tsg101* from *Camk2a*-expressing neurons led to severe, progressive neurodegeneration. Within 1 week of *Tsg101* deletion, significant GFAP staining was observed around the granule cell layer of the dentate gyrus and remained most pronounced in this region through all stages examined. Astrogliosis in the dentate gyrus would disrupt its ability to filter information passing through to the CA3 region of the hippocampus from the entorhinal cortex, which can lead to increased neuronal activity in the CA3 due to reduced recycling of glutamate and potassium from the synaptic cleft by activated astrocytes [60,61,62,63,64]. Hippocampal CA3 neurons showed caspase-dependent apoptosis within two weeks of deleting *Tsg101*, consistent with astrogliosis in the dentate gyrus leading to excitotoxicity and apoptosis in the CA3. Although embryonic fibroblasts showed a transient upregulation of autophagy prior to apoptosis following the conditional deletion of *Tsg101* [65], we did not detect lipidated LC3 (LC3-II) in the *Tsg101^ck2-null^* brains 1 week after tamoxifen treatment was initiated. This suggests that in vivo, either autophagy is not upregulated in neurons as a result of loss of *Tsg101* or it occurs early and is very brief. The presence of reactive astrocytes in the dentate gyrus may explain the difference in cell death pathways triggered in neurons in culture and neurons in vivo. Reactive astrogliosis in the dentate gyrus could reflect either local effects of loss of *Tsg101* from neurons in this region or disruption of neuronal signaling in the entorhinal cortex, which projects to and can induce astrocyte activation in the dentate gyrus. The entorhinal cortex is believed to play a role in the early stages of AD and often shows the earliest histopathological alterations, including the presence of neurofibrillary tangles and cell death [66]. We hypothesize that CA neurons may be the earliest to die in *Tsg101^ck2-null^* mice because the dentate gyrus provides excitatory inputs to the CA3, and impaired glutamate uptake by activated astrocytes in the dentate gyrus would be predicted to lead to excitotoxicity and apoptosis in CA3 neurons.

Endosomal abnormalities are some of the earliest pathological features of AD, characterized by enlarged RAB5-positive early endosomes and RAB7-positive late endosomes, as well as progressive accumulation of MVBs, lysosomes, and autophagosomes [4,9,67]. APP is known to traffic through the endosomal system, and the accumulation of APP or its N-terminal proteolytic derivatives in *Tsg101^ck2-null^* hippocampal neurons supports a role for the ESCRT pathway in normal APP trafficking. Our in vivo results are consistent with in vitro studies that demonstrated inhibited sorting of APP onto ILVs and to the lysosome, with intracellular accumulation of Aβ and a concomitant decrease in Aβ secretion when *Tsg101* was depleted [27]. It is not clear whether the accumulation of APP in *Tsg101^ck2-null^* neurons was due to reduced lysosomal degradation or impaired exosomal release, as both can be MVB-dependent. Exosomes isolated from the mouse brain are enriched with toxic APP fragments [42], suggesting exosomal secretion may clear them from cells but also deposit them into the extracellular space, where they may contribute to Aβ plaques. Mice homozygous for the human apolipoprotein E4 (ApoE4) variant, which is the greatest genetic risk factor for AD in people, showed an age-dependent decrease in exosomes associated with reduced levels of TSG101 and RAB35 [68], both of which are involved in exosome biosynthesis and release [25,69]. The role of exosomes and other EVs in AD pathogenesis remains to be fully elucidated.

Generally, ESCRT dysfunction is associated with neurodegeneration. Mutations in the gene encoding the ESCRT-III component *charged multivesicular body protein 2B *(CHMP2B) are associated with frontotemporal dementia (FTD), FTD with motor neuron disease, and amyotrophic lateral sclerosis (ALS) [70,71,72]. Studies using transgenic mice suggest *Chmp2b* mutations act through a gain-of-function mechanism [73,74]. Deleting the gene encoding the ESCRT-0 protein, HGF-regulated tyrosine kinase substrate (HGS; formerly referred to as HRS), from *Synapsin-1*-expressing neurons in mice resulted in apoptotic loss of hippocampal CA3 neurons and reduced locomotor activity and learning ability [75], although their phenotype was less severe than that observed in *Tsg101^ck2-null^* mice. Deleting *Hgs* from mouse forebrain neurons using a constitutive Camk2a-cre resulted in a phenotype more similar to what we report here for *Tsg101^ck2-null^* mice, including a marked accumulation of ubiquitinated proteins by 5 weeks of age and apoptotic and necrotic losses of hippocampal neurons by 7 weeks of age, with the CA3 region being most severely affected [76]. We have also demonstrated that deleting *Tsg101* from Schwann cells in the mouse peripheral nervous system resulted in severe, rapid onset peripheral neuropathy associated with “onion bulb” formations and de- or dysmyelination [77]. Mice lacking *Hgs* in Schwann cells displayed a much milder phenotype, displaying mild motor and sensory defects, fewer myelinated axons, and thinner and aberrantly folded myelin sheaths in the sciatic nerve [78]. Our group also previously demonstrated that the conditional ablation of *Tsg101* in oligodendroglia of the central nervous system (CNS) resulted in severe and rapid-onset spongiform neurodegeneration and de- or dysmyelination [79]. In the same study, we showed that oligodendroglial deletion of the gene encoding RAB7 did not produce any histopathological abnormalities, suggesting that the severe phenotypes associated with TSG101 deficiency were not due to defects in late endosomal, lysosomal, or autophagosomal functions, which are RAB7-dependent [80,81,82]. Taken together, these studies demonstrate critical roles for TSG101 in a variety of nervous system cell types.

Although HGS interacts with TSG101 to recruit ESCRT-I to endosomes [83,84,85], in multiple mammalian cell lines, the knockdown of TSG101 or HGS had significantly different effects on epidermal growth factor receptor (EGFR) trafficking and degradation, and endosomal and MVB morphology [65,83,84,86,87]. Depleting TSG101 resulted in the formation of multicisternal early endosomes and defects in protein sorting, as well as endoplasmic reticulum (ER) stress, ER membrane remodeling, and the upregulation of autophagy [65,86,87,88,89]. Downregulation of TSG101 has also been reported to result in reduced secretion of exosomes [25,90]. Depleting HGS did not induce early endosome tubulation and led to the production of enlarged MVBs containing fewer ILVs [18,86,88]. Thus, TSG101 appears to be required for the formation of stable vacuolar domains within the early endosome that subsequently develop into MVBs, while HGS is more important for the formation or accumulation of ILVs within MVBs. TSG101 deletion also disrupts the recycling of membrane proteins by decoupling early endosomes from recycling endosomes [23]. Given their roles in MVB production, it is not surprising that HGS and TSG101 are both involved in exosome production, with HGS depletion resulting in smaller exosomes and TSG101 depletion leading to fewer exosomes and a concomitant increase in other EV subtypes [25,86].

## 5. Conclusions

The phenotype we observed in *Tsg101^ck2-null^* mice validated some findings from cell culture studies, such as the intracellular accumulation of APP or its proteolytic derivatives in *Tsg101*-depleted neurons. Other findings in the whole brain contrasted with observations in cell culture models. For example, we did not observe lipidated LC3 in vivo within 1 week of *Tsg101* deletion, which was prior to detecting autophagy in hippocampal CA3 neurons, suggesting that if autophagy was upregulated in vivo, it was not sustained. Assessing neuronal depletion of *Tsg101* in the context of the whole brain also provided important insight into the influence of neuronal connectivity in disease pathogenesis and progression. Although all *Camk2a*-expressing forebrain neurons lost *Tsg101*, hippocampal CA3 pyramidal neurons were the first to undergo apoptosis and showed a more robust accumulation of ubiquitinated proteins and p62/SQTM1 by the 2-week time-point, whereas the dentate gyrus showed the earliest and consistently most pronounced reactive astrocytosis. We hypothesize that depleting *Tsg101* from neurons in the dentate gyrus disrupted neurotrophic signaling, triggering reactive astrogliosis to support neuronal survival and repair. The ability of activated astrocytes to execute normal functions, such as the uptake of excess glutamate from the synaptic cleft, would be impaired, and we propose that this leads to excitotoxicity and apoptosis in CA3 neurons. Our results stress the importance of in vivo studies to fully assess gene and protein functions, especially for cells that contribute to organs such as the brain, where communication between cells and different cell types is crucial.

These studies provide important proof-of-concept data toward the elucidation of complex neurodegenerative pathways in diseases such as AD. Endolysosomal abnormalities have been noted in AD-affected brain tissue and are hypothesized to contribute to the pathology of AD. Our results lend support to this hypothesis by demonstrating that impairment of the endo-lysosomal sorting machinery at TSG101 is sufficient to induce neurodegeneration in the hippocampal neurons that are most susceptible in AD, as well as to recapitulate a subset of features of AD pathology, including the accumulation of endosomal markers and Aβ. Finally, these studies demonstrate that TSG 101-dependent trafficking in neurons is critical for maintaining neuronal homeostasis.

## Data Availability

The original contributions presented in this study are included in the article/Supplementary Materials. Further inquiries can be directed to the corresponding author.

## Supplementary Materials

The following supporting information can be downloaded at: https://www.mdpi.com/article/10.3390/biom15060786/s1, Figures S1–S3: Supporting images for western blots shown in Figure 1C; Figures S4–S7: Supporting original images for western blots shown in Figure 5L; Figures S8–S10: Original confocal images for APP immunofluorescence and DAPI staining on control brains shown in Figure 6G. Figures S11–S13: Original confocal images for APP immunofluorescence and DAPI staining on *Tsg101^ck2-null^* brains shown in Figure 6H.

## Abbreviations

The following abbreviations are used in this manuscript:

TSG101	Tumor susceptibility gene 101
ESCRT	Endosomal sorting complex required for transport
p62/SQTM1	Sequestosome-1
RAB5	Ras-related protein 5
RAB7	Ras-related protein 7
LC3	Microtubule-associated protein 1 light chain 3 beta (MAP1LC3B, ATG8)
CA3	Cornu Ammonis subfield 3 of the hippocampus
AD	Alzheimer’s disease
Ab	A-beta or amyloid beta (proteolytic product of APP)
p-tau	Phosphorylated microtubule-associated protein tau (MAPT)
GTPase	Guanosine triphosphatase
APP	Amyloid precursor protein
APP-bCTF	APP β-cleavage C-terminal fragment (precursor to Aβ)
MVB	Multivesicular body
ILV	Intraluminal vesicle
EV	Extracellular vesicle
PCR	Polymerase chain reaction
Camk2a	Calcium/calmodulin-dependent protein kinase type II subunit alpha
Cre/ERT2	Tamoxifen inducible Cre recombinase
IHC	Immunohistochemistry
HRP	Horseradish peroxidase
DAB	3,3′-Diaminobenzidine
RIPA	Radioimmunoprecipitation assay buffer
TTBS	Tween Tris-buffered saline
CC3	Cleaved caspase 3
GFAP	Glial fibrillary acidic protein
NeuN	Neuronal nuclear antigen
4G8	Antibody reactive to amino acids 17–24 of APP and Aβ
CNS	Central nervous system
DNA	Deoxyribonucleic acid
H&E	Hematoxylin and eosin
TUJ1	Tubulin, beta 3 class III (TUBB3)
