# Supplementary material for: Neuronal Deletion of Tumor Susceptibility Gene 101 (Tsg101) Causes Rapid Apoptotic Loss of Hippocampal CA3 Neurons
Source: Biomolecules. 2025 May 28;15(6):786. doi: 10.3390/biom15060786 (PMC12191344; doi:10.3390/biom15060786)
Supplement: Supplementary file 1 [file biomolecules-15-00786-s001.zip › Supplemental files/Key to Supplemental material files.pdf]

Key to Supplemental material files:

Western blots
Folder 1: Files for TSG101 western blot in Figure 1. These blots were run in 2010. The electronic files are no longer available, and the images printed and entered into Will Walker's notebook were cut down. I have included snapshots from his lab notebook showing the data, and have included a TSG101 western run prior to those included in the manuscript where the wells were not well differentiated but you can see that difference in signal and the entire membrane is shown for TSG101. The GAPDH blot in the manuscript is included in full with ladder bands indicated. The mice are no longer on the shelf and we do not have any more brain samples or protein lysates to repeat the westerns, so I hope this will suffice. Ultimately, the phenotype is robust so it *Tsg101* "must" have been deleted.

Folder 2: Uncropped images for the LC3 and TUJ1 blots (Figure 5). Following imaging for LC3, the same blot was subjected to staining with anti-TUJ1.

Folder 3: Files for Figure 6G (APP on control hippocampus)

Folder 4: Files for Figure 6H (APP on $Tsg101^{ck2\text{-}null}$ hippocampus)
