# Supplementary figures and images for: Neuronal Deletion of Tumor Susceptibility Gene 101 (Tsg101) Causes Rapid Apoptotic Loss of Hippocampal CA3 Neurons
Source: Biomolecules. 2025 May 28;15(6):786. doi: 10.3390/biom15060786 (PMC12191344; doi:10.3390/biom15060786)

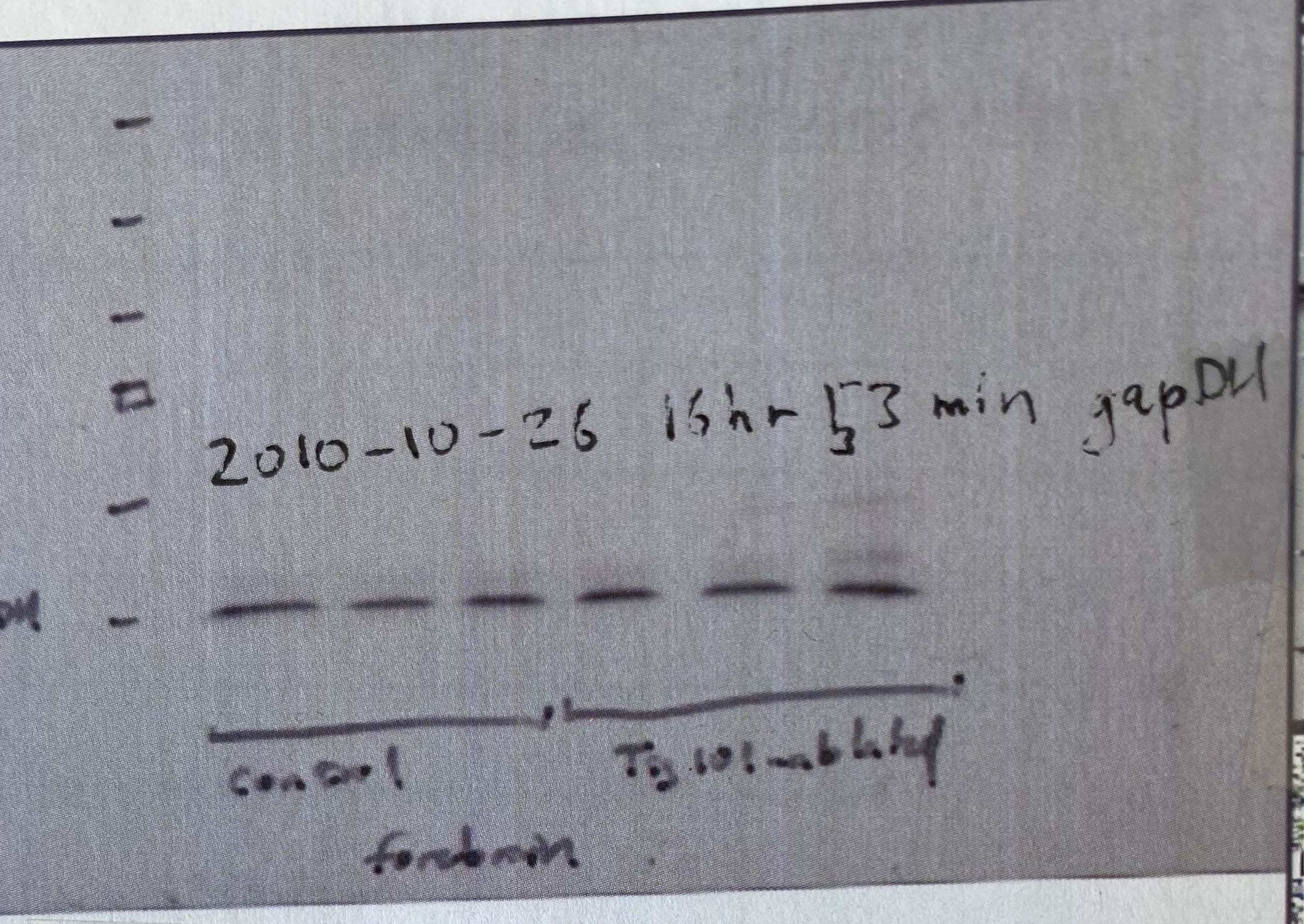

Supplement: Supplementary file 1 [file biomolecules-15-00786-s001.zip › Supplemental files/Folder 1 TSG101 WB/Figure S1 GAPDH forebrain.png]

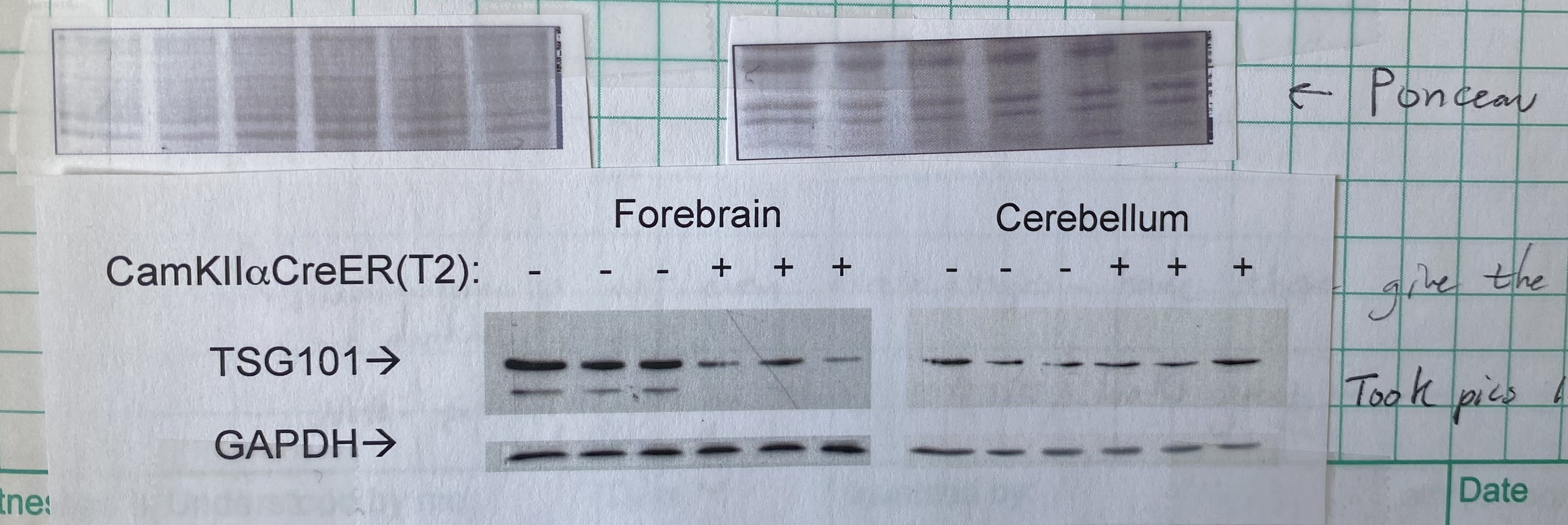

Supplement: Supplementary file 1 [file biomolecules-15-00786-s001.zip › Supplemental files/Folder 1 TSG101 WB/Figure S2 TSG101 and ponceau forebrain and cerebellum.png]

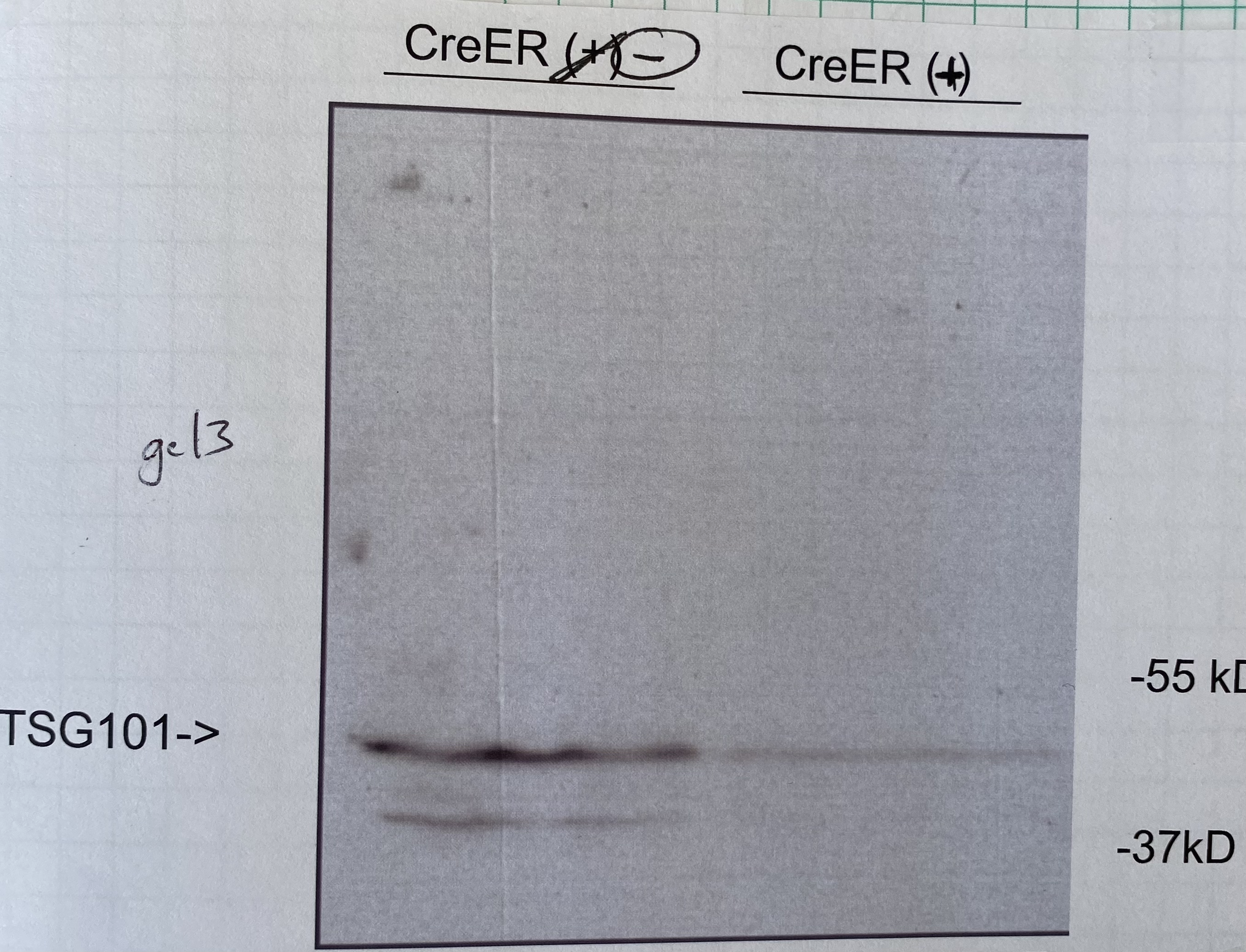

Supplement: Supplementary file 1 [file biomolecules-15-00786-s001.zip › Supplemental files/Folder 1 TSG101 WB/Figure S3 TSG101 full size not in paper.png]

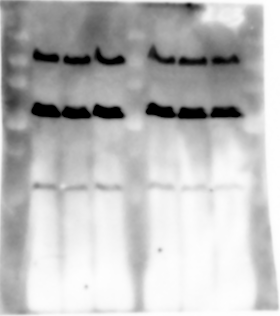

Supplement: Supplementary file 1 [file biomolecules-15-00786-s001.zip › Supplemental files/Folder 2 LC3 WB/Figure S4 lc3 and tuj1 wb.tif]

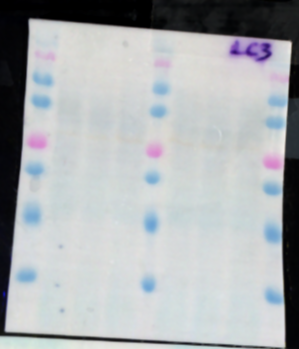

Supplement: Supplementary file 1 [file biomolecules-15-00786-s001.zip › Supplemental files/Folder 2 LC3 WB/Figure S5 lc3 only blot ladder.tif]

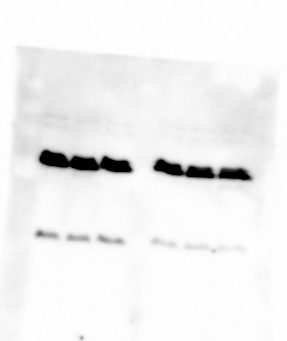

Supplement: Supplementary file 1 [file biomolecules-15-00786-s001.zip › Supplemental files/Folder 2 LC3 WB/Figure S6 lc3 only.tif]

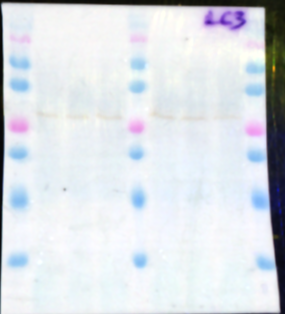

Supplement: Supplementary file 1 [file biomolecules-15-00786-s001.zip › Supplemental files/Folder 2 LC3 WB/Figure S7 lc3 tuj1 ladder.tif]

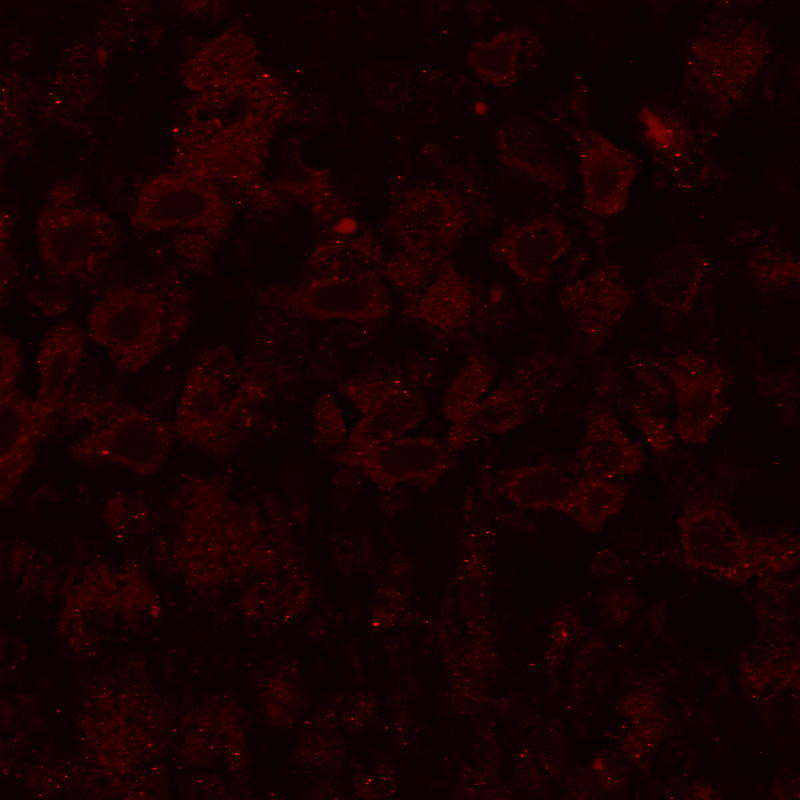

Supplement: Supplementary file 1 [file biomolecules-15-00786-s001.zip › Supplemental files/Folder 3 APP IF control/Figure S10 Tsg101 ckII oil 2 weeks APPred hippocamp 40X x2-1_C002.tif]

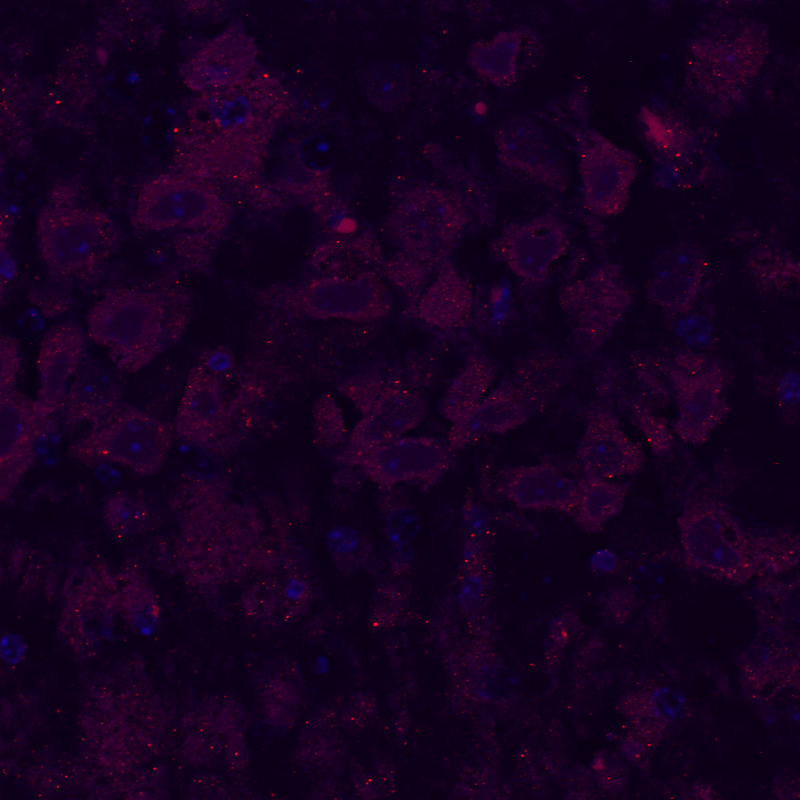

Supplement: Supplementary file 1 [file biomolecules-15-00786-s001.zip › Supplemental files/Folder 3 APP IF control/Figure S8 Tsg101 ckII oil 2 weeks APPred hippocamp 40X x2-1_.tif]

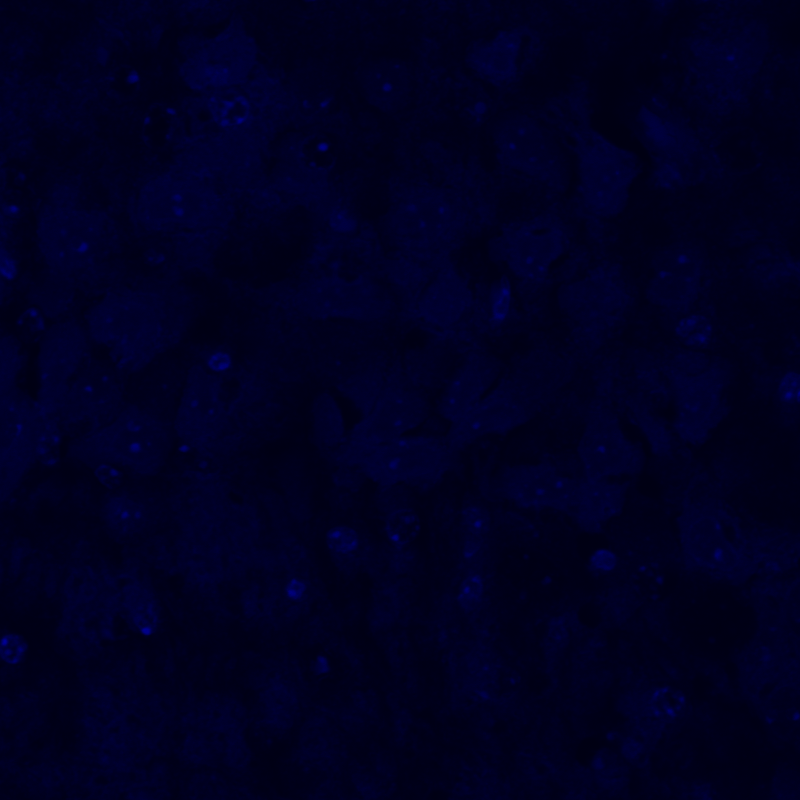

Supplement: Supplementary file 1 [file biomolecules-15-00786-s001.zip › Supplemental files/Folder 3 APP IF control/Figure S9 Tsg101 ckII oil 2 weeks APPred hippocamp 40X x2-1_C001.tif]

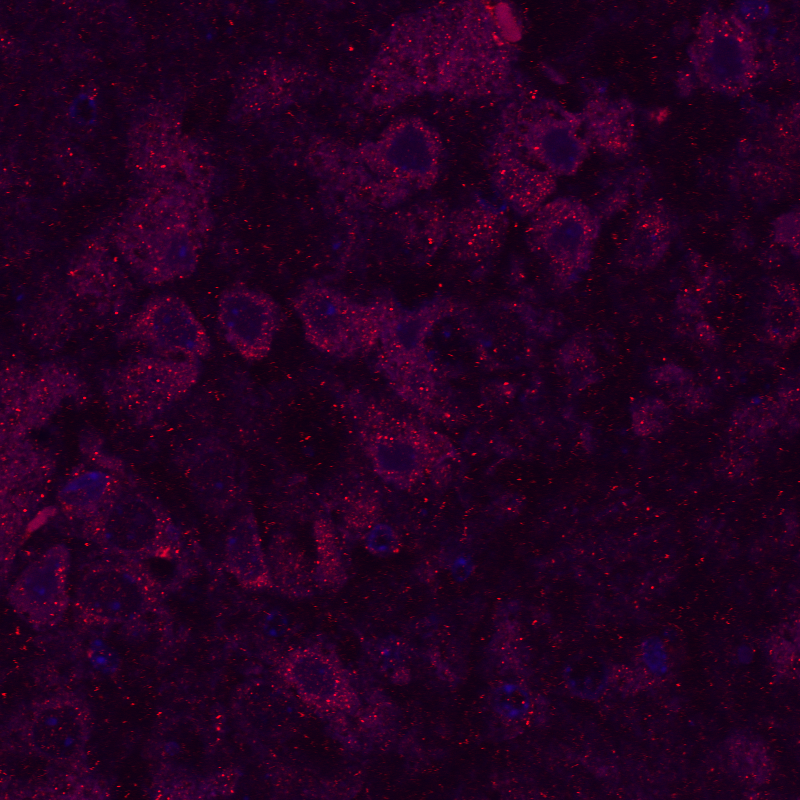

Supplement: Supplementary file 1 [file biomolecules-15-00786-s001.zip › Supplemental files/Folder 4 APP IF Tsg101ck2-null/Figure S11 Tsg101 ckII tam 2 weeks APPred hippocamp 40X x2-4_.tif]

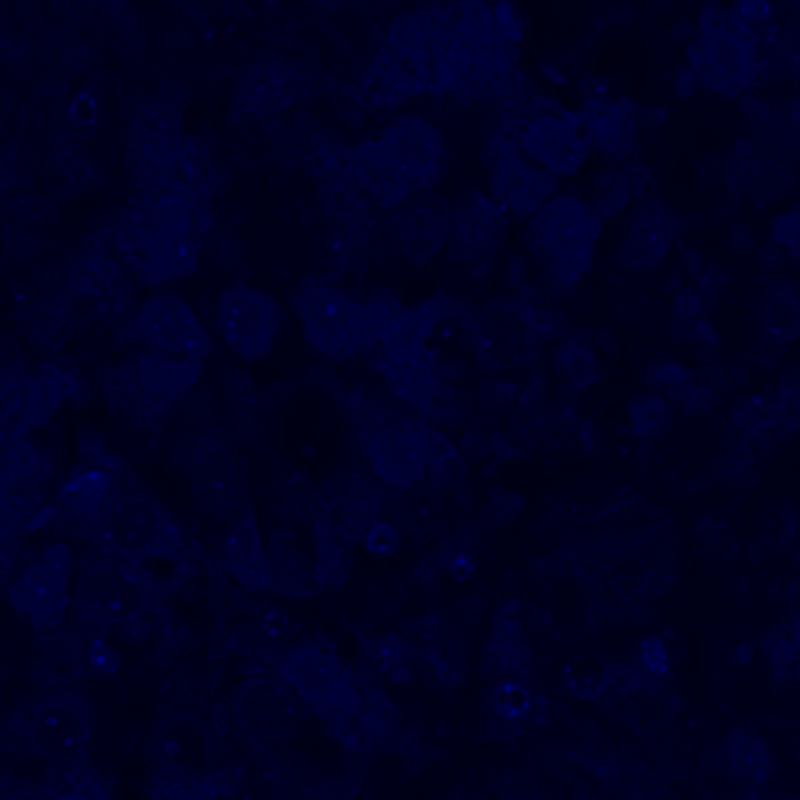

Supplement: Supplementary file 1 [file biomolecules-15-00786-s001.zip › Supplemental files/Folder 4 APP IF Tsg101ck2-null/Figure S12 Tsg101 ckII tam 2 weeks APPred hippocamp 40X x2-4_C001.tif]

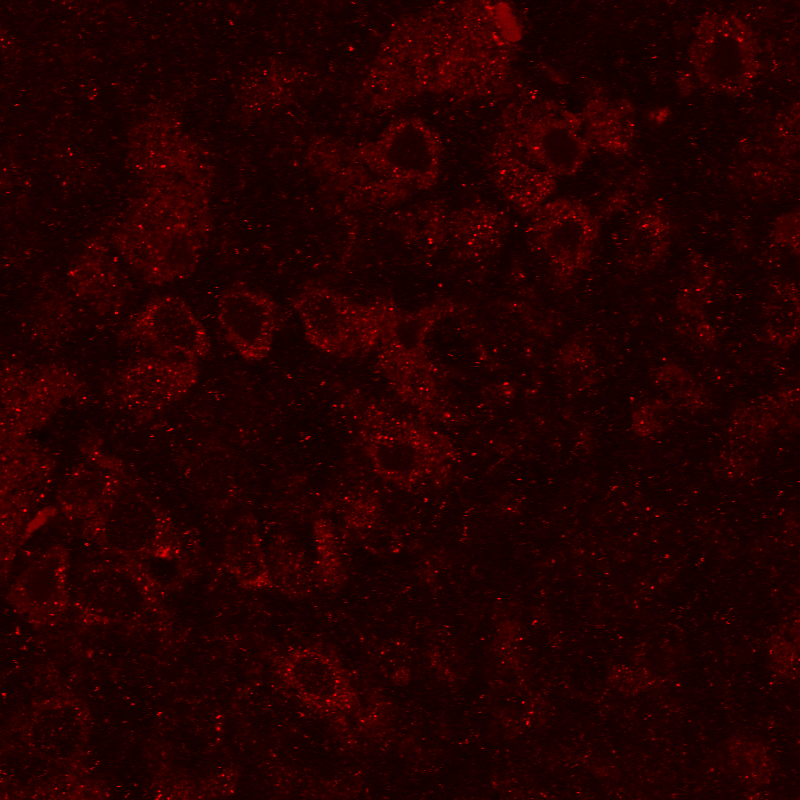

Supplement: Supplementary file 1 [file biomolecules-15-00786-s001.zip › Supplemental files/Folder 4 APP IF Tsg101ck2-null/Figure S13 Tsg101 ckII tam 2 weeks APPred hippocamp 40X x2-4_C002.tif]
